# PLCG2 as a potential indicator of tumor microenvironment remodeling in soft tissue sarcoma

**DOI:** 10.1097/MD.0000000000025008

**Published:** 2021-03-19

**Authors:** Zhengtian Li, Rong Zhao, Wenkang Yang, Chan Li, Jun Huang, Zhenpei Wen, Gang Du, Lingling Jiang

**Affiliations:** a Guangxi Medical University; b Department of Bone and Joint Surgery, The First Affiliated Hospital of Guangxi Medical University, Nanning; c Department of Anesthesiology, The second Hospital of Anhui Medical University, Hefei, China.

**Keywords:** immune cells, prognosis, soft tissue sarcoma, the cancer genome atlas, tumor microenvironment, tumor microenvironment remodeling

## Abstract

The tumor microenvironment (TME) plays an important role in the occurrence and development of soft tissue sarcoma (STS). A number of studies have shown that to inhibit tumor growth, the TME can be remodeled into an environment unsuitable for tumor proliferation. However, a lack of understanding exists regarding the dynamic regulation of TME.

In this study, we used CIBERSORT and ESTIMATE calculation methods from the Cancer Genome Atlas (TCGA) database to calculate the proportion of tumor infiltrating immune cells (TICs) and the number of immune and stromal components in 263 STS samples. Differential expression genes (DEGs) shared by Immune Score and Stromal Score were obtained via difference analysis. Univariate Cox regression analysis and construction of protein–protein interaction (PPI) networks were applied to the DEGs.

Through intersection analysis of univariate COX and PPI, PLCG2 was determined as the indicator. Further analysis showed that PLCG2 expression was positively correlated with the survival of STS patients. Gene set enrichment analysis (GSEA) showed that genes in the highly expressed PLCG2 group were enriched in immune-related activities. In the low-expression PLCG2 group, genes were enriched in the E2F, G2M, and MYC pathways. Difference analysis and correlation analysis showed that CD8^+^ T cells, gamma delta T cells, monocytes, and M1 macrophages were positively correlated with PLCG2 expression, indicating that PLCG2 may represent the immune status of TME.

Therefore, the level of PLCG2 may aid in determining the prognosis of STS patients, especially the status of TME. These data provide additional insights into the remodeling of TME.

## 1 Introduction

Soft tissue sarcoma (STS) is a rare and heterogenous mesenchymal neoplasm. It is estimated that there were 13,040 newly diagnosed STS cases and 5150 deaths in the United States in 2018.^[1]^ STS is a complex disease that includes more than 50 histological subtypes, which can occur throughout the body including the limbs, trunk, and retroperitoneum.^[2]^ Owing to the diversity of STS, some patients exhibit obvious symptoms where other cases are discovered unintentionally. Thus, imaging and pathology play particularly important roles in the early diagnosis of STS. Localized STS can be effectively treated with surgery. However, some patients present with metastatic disease at initial diagnosis; their median overall survival (OS) is 14 months despite comprehensive management.^[3]^ Therefore, there is an urgent need to explore the carcinogenesis and therapeutics of STS.

The tumor microenvironment (TME) is defined as the internal and external environment in which tumor cells exist. It is composed of surrounding blood vessels, other non-malignant cells, the extracellular matrix, and signaling molecules. The TME not only plays a pivotal role in tumorigenesis, progression, and metastasis, but also has a significant effect on treatment efficacy.^[4–7]^ Stromal cells, as core members of the TME, facilitate the proliferation of pancreatic cancer cells and build a barrier to reduce the efficacy of chemotherapeutic drugs.^[8]^ It is well known that the microenvironment is initially involved in curbing the growth of the tumor and protecting the survival of normal cells. However, as the tumor cells continue to proliferate, the microenvironment is gradually transformed into a suitable milieu for the survival of tumor cells. A growing body of studies have shown that to inhibit tumor growth, the TME can be remodeled into an unsuitable environment for tumor cell proliferation.^[9,10]^ At present, current therapies targeting the TME are used in clinical practice such as angiogenesis inhibitors and aromatase inhibitors (applied in breast cancer).^[11]^ Therefore, TME remolding may be crucial to effectively inhibiting STS progression, However, there is relatively little research in this area.

Tumor-infiltrating immune cells (TICs), which are the main immune components in the TME, are immune cells that migrate from the blood to the tumor tissues. Normally, immune cells recognize and trap abnormal cells in the body. However, in the TME, immune cells may instead aid tumor cells in escaping immune surveillance. The struggle between cancer and immune cells occurs in 3 stages: elimination, equilibrium, and escape.^[12,13]^ With deeper study of tumor immune mechanisms, it has been found that TICs are closely correlated with clinical outcomes and may therefore be used as a drug target to improve patient survival.^[14–16]^ For example, TICs in lung cancer may be used to determine both prognosis and response to immunotherapies.^[17]^ Therefore, determination of immune status in TME may have an important impact on the prognosis and treatment of tumor patients, and it may play a role in the remodeling of the TME.

The aim of this study was to identify an indicator for the status of the TME via bioinformatics analysis. In this study, we analyzed differentially expressed genes (DEGs) generated via comparison between immune components and stromal components in STS samples from the Cancer Genome Atlas (TCGA). Results revealed that PLCG2 may be a potential indicator of TME remodeling and may be involved in curbing STS tumor growth.

## 2 Material and methods

### 2.1 Raw data access

We downloaded transcriptome data and the corresponding clinical data of STS patients from the TCGA database (http://cancergenome.nih.gov/).

### 2.2 Generation of TME score

The TME score of each sample was generated using the “Estimate” package in R software (version 4.0.0; https://www.r-project.org/). We used 3 different scores (Stromal Score, Immune Score, and Estimate Score) to represent the proportions of stromal cells, immune cells, and the sum of these 2 TME components. The higher the score, the greater the amount in the TME.

### 2.3 TME component-related survival analysis

We divided 259 samples into a high-score subgroup and a low-score group according to the median value of the 3 scores and performed survival analysis. The survival curve was drawn using the Kaplan–Meier method, and log-rank test was utilized to determine statistical significance. A *P* < .05 was considered statistically significant.

### 2.4 Correlation analysis of TME components and STS patient gender

We performed correlation analysis to determine correlation between the proportion of TME components and gender. A *P* < .05 was considered statistically significant.

### 2.5 TME component-related difference analysis of gene expression

We performed difference analysis for gene expression in the stromal and immune components via the “limma” package in R. The genes that met the requirement of |logFC|>1 and had a *P* value <.05 (adjusted by false discovery rate [FDR]) were considered differentially expressed. Genes that were differentially expressed in the stromal and immune components were intersected to identify the final differentially expressed genes (DEGs).

### 2.6 Functional enrichment analysis

Both gene ontology (GO) analysis and Kyoto Encyclopedia of Genes and Genomes (KEGG) pathway enrichment analyses were used to investigate the impact of DEGs via the “clusterProfiler,” “org.Hs.eg.db,” “enrichplot” and “ggplot2” packages in R.

### 2.7 PPI network establishment and key DEGs identification

The STRING database (https://string-db.org/) was utilized to identify protein interaction networks based on above DEGs (interaction score=0.95), and the PPI network plot was drawn using Cytoscape software (version 3.7.2; https://cytoscape.org/). Node counts were used as evaluation criterion to identify 30 key DEGs from the PPI network.

### 2.8 COX regression analysis

We performed univariate COX analysis among the DEGs via the “survival” package in R. The resultant prognostic genes (*P* < .01) were added into an intersection analysis with the key DEGs chosen from the PPI network to identify the final key gene.

### 2.9 Gene set enrichment analysis (GSEA)

We utilized GSEA software (version 4.0.3; https://www.gsea-msigdb.org/gsea/) in the Hallmark gene sets to investigate the functional pathway in which the final key gene may be enriched. Gene sets were considered significant only when their nominal *P* value was <.05 and the FDR q value was <0.05.

### 2.10 Tumor infiltrate immune cell profile

We assessed the relative quantity of immune cells in tumor samples by applying CIBERSORT, a complete immune cell estimation analysis algorithm. *P* < .05 suggested an accurate estimation.

## 3 Results

### 3.1 TME score generation and related survival analysis

**Figure 1:**
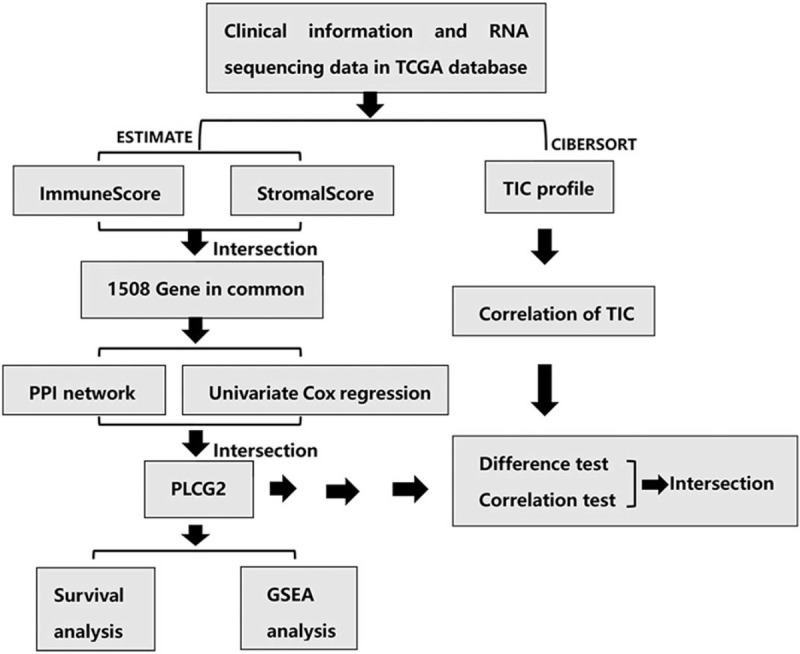
Study workflow.

**Figure 2:**
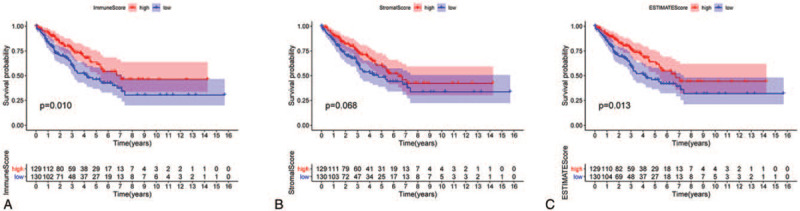
Correlation of scores with the survival of STS patients. (A) Survival analysis of STS patients grouped into high or low score groups in Immune Score as determined by comparison with the median (*P* = .010). (B) Survival curve of Stromal Score with *P* = .068. (C) Survival analysis of Estimate Score with *P* = .0.13. STS = soft tissue sarcoma.

**Figure 3:**
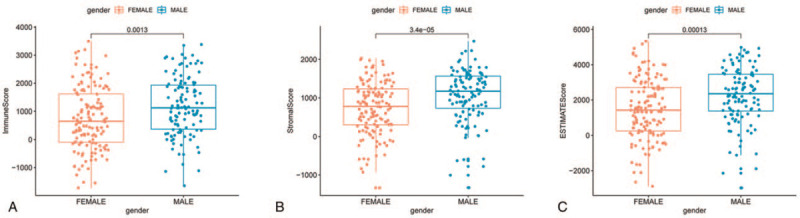
Correlation of Immune Score, Stromal Score, and Estimate Score with gender.

The workflow of this study is shown in Figure 1. Based on the transcriptome data from 263 STS samples (total samples: 265; normal samples: 2; tumor samples: 263), we calculated the proportions of TME components and applied 3 scores (Stromal Score, Immune Score, and Estimate Score) to represent the proportions of stromal cells, immune cells, and the sum of the 2 components, respectively. The higher the score, the greater the amount of the corresponding component in the TME. After the 263 samples were scored, they were grouped by the mean values of the 3 scores. The results were then added to the survival analysis from the STS patient survival data. K–M curves of the 3 survival analyses are shown in Figure 2. The results of the log-rank test indicated that both the Estimate Score (Fig. 2C) and the Immune Score (Fig. 2A) considerably influenced the OS of patients while the Stromal Score (Fig. 2B) did not. These results implied that immune components in the TME were suitable for indicating the prognosis of STS patients. Interestingly, correlation analysis showed that all 3 scores were clearly related with the gender of STS patients. This indicates that the above scores of male patients were significantly different from those of the female patients (*P* < .05, Fig. 3A–C).

### 3.2 TME score-related gene expression difference analysis

**Figure 4:**
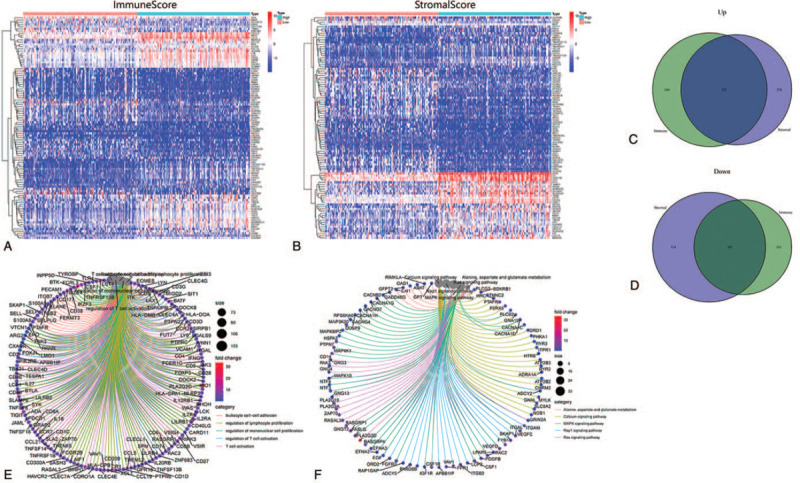
Heatmaps, Venn plots, GO enrichment analysis, and KEGG of DEGs. (A, B) Heatmap of DEGs generated by comparison of the high score group vs the low score group in Immune Score and Stromal Score, respectively. (C, D) Venn plots showing common up-regulated and down-regulated DEGs shared by Immune Score and Stromal Score. (E, F) GO and KEGG enrichment analysis of DEGs. DEGs = differentially expressed genes, GO = gene ontology, KEGG = Kyoto Encyclopedia of Genes and Genomes.

The comparison between the high Stromal Score subgroup and the low score group screened 999 upregulated genes and 1517 downregulated genes. Similarly, the difference analysis of the high vs low Immune Score subgroups screened differentially expressed genes, including 1065 upregulated genes and 1134 downregulated genes. A heatmap of the genes is shown in Figure 4A, B. As shown by the Venn diagram (Fig. 4C, D), difference analyses between the high and low score subgroups from the Immune Score and Stromal Score subgroups also co-identified 1508 differently expressed genes (DEGs).

### 3.3 Enrichment analysis

GO and KEGG enrichment analyses were used to investigate the tumor-related mechanisms of the DEGs. Significant enrichment of DEGs in immune-related GO terms (i.e., leukocyte cell-cell adhesion and T-cell activation) were observed (Fig. 4E). The KEGG enrichment analysis illustrated that DEGs were abundantly enriched in some well-known pathways that play important roles in regulating cell proliferation, growth, and differentiation, such as the Ras, MAPK, and calcium signaling pathways (Fig. 4F). This suggests that DEGs play a role in immune function. More detailed results are shown in the supplementary materials. Thus, the overall function of the DEGs were mapped to immune-related activities, implying that the involvement of immune factors is a predominant feature of the TME in STS.

### 3.4 PPI network and univariate COX regression

**Figure 5:**
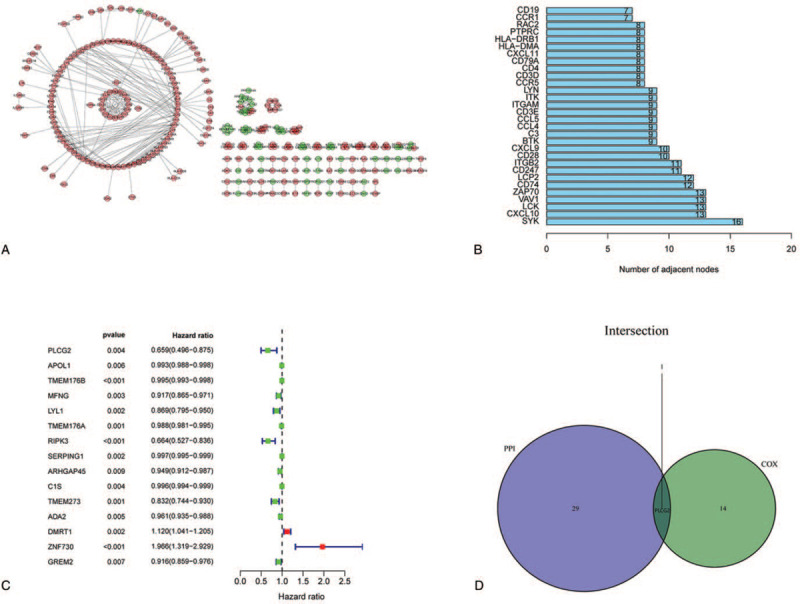
Protein–protein interaction network and univariate COX regression analysis. (A) Interaction network constructed with the nodes from the interaction confidence value >0.95. (B) The top 30 genes ordered by the number of nodes. (C) Univariate COX regression analysis with 1,508 DEGs (*P* < .01). (D) Venn plot showing the common factors shared by 30 leading nodes in the PPI and top significant factors from the univariate COX regression.

Based on the 1508 DEGs, we constructed a protein–protein interaction network (consisting of 285 nodes and 408 edges) and a PPI plot (Fig. 5A) drawn using Cytoscape software. Genes that contributed to the PPI were sorted by number of nodes, as shown in Figure 5B. In addition, we performed univariate Cox regression analysis (Table 1) using survival information from the STS patients and identified 15 prognostic genes. The resultant genes are displayed in Figure 5C. To further identify the key gene, results of the 2 above analyses were intersected and ultimately, the PLCG2 gene was selected (Venn diagram, Fig. 5D).

**Table 1:** Univariate Cox regression analysis results.

Gene	KM	HR	HR.95L	HR.95H	*P* value
PLCG2	0.00899	0.65859	0.49587	0.87470	.00391
APOL1	0.00789	0.99289	0.98791	0.99790	.00550
TMEM176B	0.00043	0.99524	0.99260	0.99788	.00042
MFNG	0.00605	0.91663	0.86495	0.97141	.00328
LYL1	0.00074	0.86906	0.79508	0.94994	.00199
TMEM176A	0.00026	0.98803	0.98073	0.99538	.00146
RIPK3	0.00133	0.66357	0.52657	0.83622	.00050
SERPING1	0.00021	0.99692	0.99502	0.99882	.00150
ARHGAP45	0.00755	0.94887	0.91222	0.98700	.00903
C1S	0.00246	0.99622	0.99365	0.99880	.00416
TMEM273	0.00658	0.83206	0.74443	0.93001	.00120
ADA2	0.00271	0.96117	0.93484	0.98824	.00520
DMRT1	0.00474	1.11982	1.04092	1.20471	.00239
ZNF730	0.00808	1.96557	1.31887	2.92935	.00090
GREM2	0.00017	0.91591	0.85938	0.97616	.00688

∗H = high, HR = hazard ratio, KM = Kaplan–Meier, L = low.

### 3.5 The correlation between PLCG2 expression and the survival of STS patients

**Figure 6:**
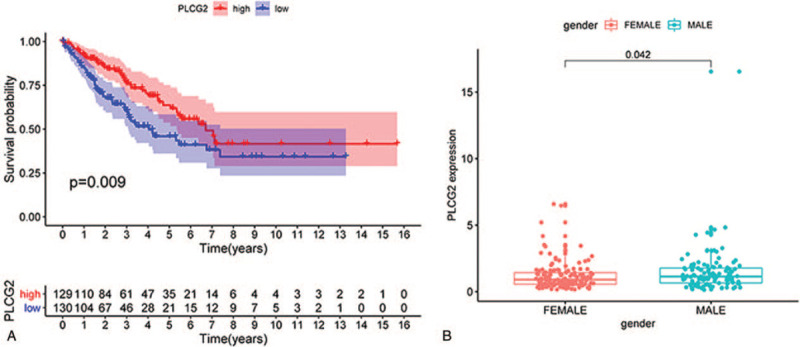
The correlation between PLCG2 expression and survival as well as gender characteristics of STS patients.

PLCG2 was selected for further research in this study. Survival analysis of 261 patients was conducted by dividing the sample into a high expression phenotype gene set and a low expression gene set using the mean expression degree of PLCG2. Results indicated that PLCG2 influenced the survival status of STS patients (Fig. 6A). Our study showed that the expression level of PLCG2 was significantly different in male and female patients (Fig. 6B). The above results clearly indicate that the expression of PLCG2 in the TME was positively correlated with the prognosis of STS patients.

### 3.6 Gene set enrichment analysis (GSEA)

**Figure 7:**
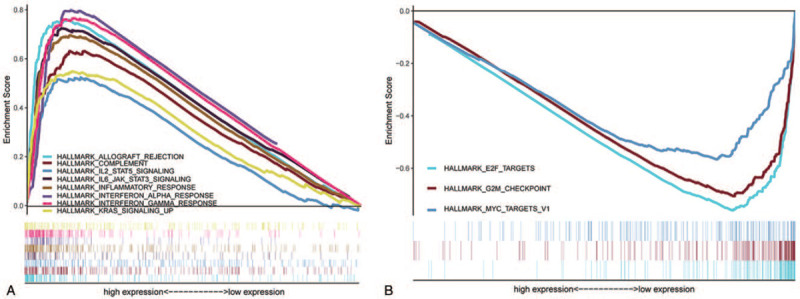
GSEA of samples with high and low PLCG2 expression. (A) The enriched gene sets in the HALLMARK collection for the high PLCG2 expression sample. Only gene sets with NOM *P* < .05 and FDR q < 0.06 were considered significant. Only the leading gene sets are displayed in the plot. (B) The enriched gene sets in the HALLMARK collection from samples with low PLCG2 expression. (C) Enriched gene sets in the C7 collection, the immunologic gene sets, from samples with high PLCG2 expression. Only the leading gene sets are shown in the plot. (D) Enriched gene sets in the C7 collection from the low PLCG2 expression group. Only the leading gene sets are shown in the plot.

GSEA performed on the 2 groups (high PLCG2 expression and low expression) showed that the genes in the PLCG2 high expression group were mainly enriched in immune-related pathway such as IL-2/STAT5 signaling pathway, IL-6/JAK/STAT3 signaling pathway and interferon response (Fig. 7A). In the PLCG2 low expression group, the genes were enriched in E2F, G2M, and MYC function gene sets (Fig. 7B). These results suggest that PLCG2 may be a potential indicator of TME status.

### 3.7 Tumor immune cell infiltration analysis

**Figure 8:**
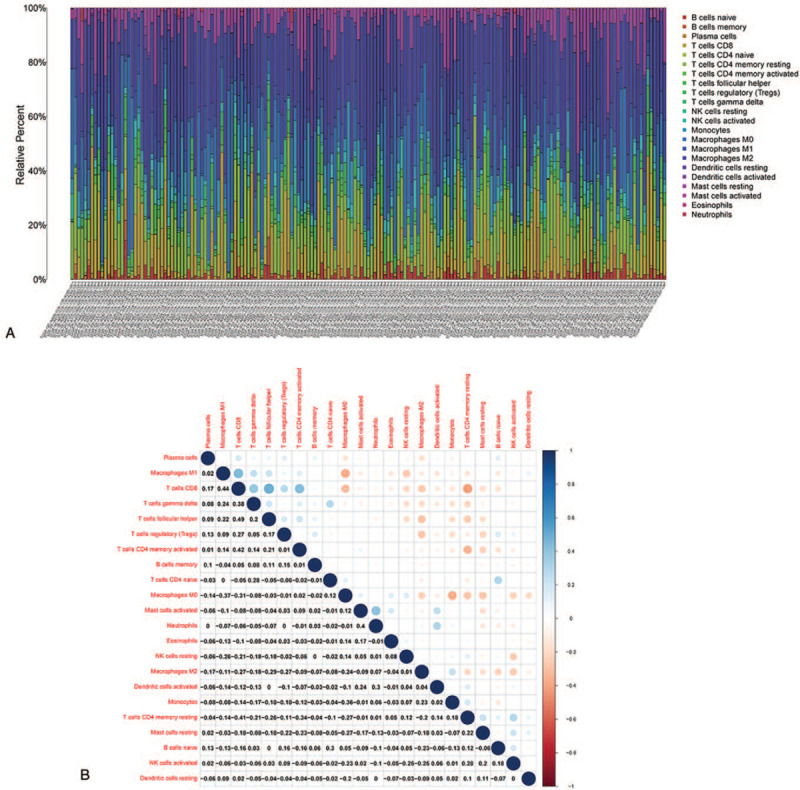
TIC profile in tumor samples and correlation analysis. (A) Bar plot showing the proportion of 22 types of TICs in STS tumor samples. (B) Heatmap showing the correlation between 22 types of TICs.

**Figure 9:**
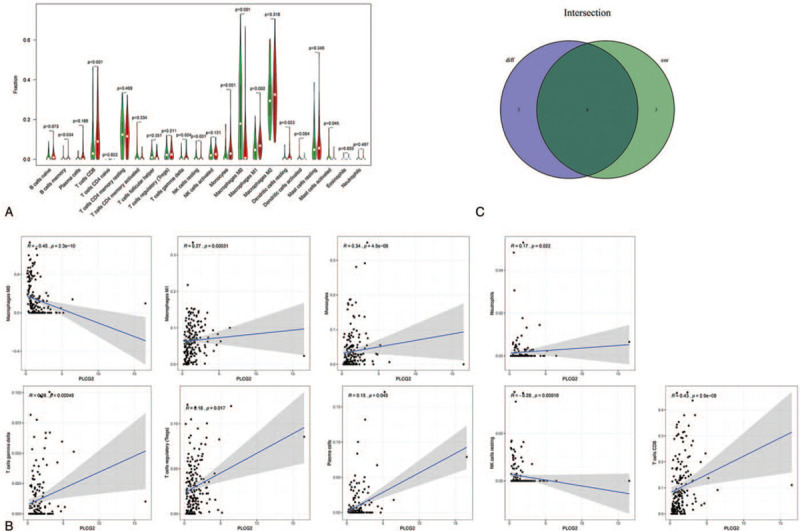
Correlation of TICs proportion with PLCG2 expression. (A) Violin plot showing the differentiation ratio of 22 types of immune cells between STS tumor samples with low or high PLCG2 expression, relative to the median PLCG2 expression level. (B) Scatter plot showing the correlation of 9 types of TICs with PLCG2 expression (*P* < .05). (C) Venn diagram displaying 6 types of TICs correlated with PLCG2 expression.

Using CIBERSORT, a complete immune cell estimation analysis method, we assessed the proportions of 22 types of immune cells in STS samples and provided a graphical view of the resultant output profiles via Barplot and Heatmap (Fig. 8A, B). We also performed difference analysis on the TICs between 2 groups (high PLCG2 expression group and low expression group); the results are shown via violin plot (Fig. 9A). Then, correction analysis of PLCG2 expression and TICs proportion was performed (Fig. 9B). The results from the difference and correlation analyses showed that a total of 6 types of TICs were correlated with the expression of PLCG2 (Fig. 9C, Table 2). Among these, 4 types of TICs were positively correlated with PLCG2 expression, including CD8^+^ T cells, monocytes, M1 macrophages, and gamma delta T cells. Additionally, 2 types of TICs were negatively correlated with PLCG2 expression, resting NK cells and M0 macrophages. These results further support the hypothesis that PLCG2 level may affect TME immune activity.

**Table 2:** Difference test and correlation test results.

Difference test	*P* value	Correlation test	*P* value
B cells memory	.034	Plasma cells	.045
T cells CD8	<.001	T cells CD8	<.001
T cells gamma delta	.024	T cells regulatory (Tregs)	.017
NK cells resting	<.001	T cells gamma delta	<.001
Monocytes	<.001	NK cells resting	<.001
Macrophages M0	<.001	Monocytes	<.001
Macrophages M1	.001	Macrophages M0	<.001
Dendritic cells resting	.022	Macrophages M1	<.001
Mast cells activated	.044	Neutrophils	.022

## 4 Discussion

The TME contains diverse cell types, which are important components of tumor tissues and play an essential role in the initiation and development of cancer. The cells and molecules within the TME change dynamically, indicating tumor characteristics and promoting immune escape, growth, and metastasis.^[18,19]^ A number of studies have shed light on the clinical significance of the TME in the prediction of treatment efficacy and patient prognosis.^[20–22]^ In recent years, multiple drugs aimed at the TME such as immune checkpoint inhibitors and angiogenesis inhibitors have demonstrated remarkable efficacy in restraining the progression and metastasis of tumors.^[23,24]^ Likewise, exploration of TME status and relevant therapies for STS has also made progress. Studies have shown that the internal components of the TME can change and that its status is transmitted by signaling pathways that influence the efficacy of antineoplastic drugs such as programmed cell death-1 (PD-1) inhibitors in STS.^[25]^ In addition, recent studies indicate that remodeling of the TME by immune checkpoint inhibitors or adoptive cell transfer exhibited satisfactory effects in reducing tumor progression and improving treatment outcomes in some types of STS.^[26,27]^ This evidence suggests that remodeling of the TME in STS is of clinical significance and deserves further study.

To further understand the TME and its dynamic process in STS, we identified an indicator of TME status that may be used to predict the prognosis of STS patients. This indicator may also be a therapeutic target that could be leveraged to convert the TME status into 1 of tumor suppression. In our study, we identified the TME-related genes that may be used to predict TME status by performing relevant bioinformatics analyses using STS data from the TCGA database.

We also calculated the proportions of TME components and performed related survival analysis. The results indicated that both the Estimate Score (*P* = .013) and the Immune Score (*P* = .010) were significantly related to the OS of STS patients while the Stromal Score (*P* = .068) was not. This implies that the immune components of the TME are relevant to the prognosis of STS patients and suitable as prognosis biomarkers. Previous studies had proved that TICs have crucial prognosis value in solid tumors, and this was influenced by type, density, and location of immune cells, consistent with our results.^[28–34]^ Further, we unexpectedly found that all 3 scores in the TME of male patients were higher than in female patients. The cause and practical significance of this result is unclear and requires additional research. Then, we identified DEGs using TME score-related gene expression difference analysis and conducted GO and KEGG enrichment analysis. The results illustrated that the DEGs were enriched in immune-related terms such as leukocyte cell-cell adhesion and T-cell activation, as well as pathways including the calcium signaling pathway, MAPK signaling pathway, and Ras signaling pathway. Therefore, hypothesize that TME-related DEGs influence the TME immune microenvironment through these signaling pathways. The DEGs were further applied to the PPI network and univariate COX regression analysis. The 2 analysis methods are mainly used to screen feature variables and build the best model. And then, the intersection analysis between the leading nodes in PPI network and the top 15 factors ranked by the *P* value of univariate COX regression was carried out, and only 1 factors, PLCG2, was overlapping from the above analysis (Fig. 5). Thus, we identified phospholipase C gamma 2 (PLCG2) as the optimal TME-related target gene via the above intersection analysis. It should be noted, however, that the PLCG2, which was selected by integrating the union of features from the above 2 analysis methods, was reliable in further validations in this study, suggesting that the integration strategy was feasible. Further analysis confirmed that PLCG2 was significantly related to OS (*P* = .009) and the gender of STS patients. PLCG2 encodes a transmembrane signaling enzyme, which catalyzes the hydrolysis of phosphatidylinositol 4, 5-bisphosphate (PIP2) into diacylglycerol (DAG) and inositol 1, 4, 5-trisphosphate (IP3), and second messenger molecules, which are vital for delivering signals from growth factor and immune system receptors across the cell membrane, with the aid of calcium.^[35]^ A risk score predicting OS was built utilizing PLCG2 and genes identified in colon cancer patients, and was correlated with the number of tumor-infiltrating immune cells.^[36]^ Furthermore, mutational analyses performed in chronic lymphocytic leukemia patients suggested that acquired ibrutinib resistance and progression was linked to mutation of the PLCG2 gene.^[37,38]^ However, studies focused on PLCG2 in other solid tumors, especially STS, are lacking. The GSEA results suggested that the high PLCG2 expression group was mainly enriched in immune-related pathways such as IL-2/STAT5 signaling pathway and IL-6/JAK/STAT3 signaling pathway, while the low PLCG2 expression group was enriched in 3 gene sets whose function is uncertain. Hence, we inferred that the reduction of PLCG2 expression was correlated with the status conversion of TME from strong immune to weak immune. The IL-6/JAK/STAT3 pathway has a key role in the growth and development of many human cancers. Multiple cell types in the TME produce IL-6, leading to activation of JAK/STAT3 signaling in both tumor cells and tumor-infiltrating immune cells, which can promote tumor-cell proliferation, survival, invasiveness, and metastasis.^[39]^ STAT3 is often hyperactivated in tumor-infiltrating immune cells and exerts negative regulatory effects on neutrophils, natural killer (NK) cells, effector T cells, and dendritic cells (DCs), suggesting that STAT3 activation in immune cells likely leads to downmodulation of anti-tumor immunity.^[40–44]^ Although several studies have linked STAT3 to tumor growth and metastases in various types of neoplasms, it was reported that STAT3 activation is also correlated with a better prognosis in some tumors, such as head and neck squamous cell carcinoma.^[45]^ STS are rare and heterogeneous mesenchymal neoplams, with more 70 histological subtypes, and various subtypes having a different prognostic.^[46]^ Management of STS is increasingly subtype-dependent. Hirofumi Bekki et al. reported that phosphorylation of STAT3 in undifferentiated pleomorphic sarcoma was correlated with a favorable prognosis.^[47]^ R Lai et al found that among 31 patients who presented with localized Ewing sarcoma, high-level STAT3 activation correlated with better OS.^[48]^ Similarity, Nokitaka Setsu et al also reported that activator of STAT3 in soft tissue leiomyosarcoma is associated with a better prognosis.^[49]^ Research had shown that high expression of PLCG2 can promote the activation of IL2 and STAT3 in TME,^[50]^ which is consistent with our GSEA results. De La Iglesia et al^[51]^ reported that STAT3 plays a pro-oncogenic or tumor suppressive role in glioblastoma depending on the mutational profile of the tumor. STAT3 seems to have a double-sided effect on tumor progression and it is possible that it functions as an oncoprotein or a tumor suppressor protein. In conclusion, PLCG2 may affect IL-6/JAK/STAT3 signaling pathway to form anti-tumor TME, which may depend on the subtype of STS and the role of STAT3 in TME. It will require more further research in the future. The above works indicated that PLCG2 might be a potential indicator of the TME status in STS patients and that TME remodeling targeted to PLCG2 may provide a strategy for suppressing tumor progression.

Tumor immune cell infiltration analysis verified the above conclusion and identified 6 types of TICs correlated with PLCG2 expression. Among these, CD8^+^ T cells, gamma delta T cells, M1 macrophages, and monocytes were positively correlated with PLCG2 expression, while resting NK cells and M0 macrophages were negatively correlated with PLCG2 expression. Previous studies using preclinical models showed the impact of CD8^+^ T cells on the suppression of tumor cell growth, tumor infiltration inhibition, and the regulation of complete tumor elimination.^[52–54]^ Similarly, several studies have indicated that gamma delta T cells can kill a wide range of tumor cells from both solid tumors and hematopoietic malignancies.^[55,56]^ Macrophages can be activated by a variety of different cytokines within the microenvironment. M1 macrophages were associated with anti-tumorigenic functions, while M2 macrophages tend to be associated with pro-tumorigenic phenotypes. M1 macrophages are considered antitumor because they contribute to the innate host defense and tumor cell destruction by producing pro-inflammatory cytokines and other substances.^[57]^ Induction of macrophage phenotype polarization from M2 macrophages to M1 macrophages facilitates TME conversion from an immune-suppressive to an immune-promoting environment, which is currently applied in the clinical therapy of tumors.^[58]^ However, our study showed a positive correlation between the expression of PLCG2 and M1 macrophages, indicating that the activation or high expression of PLCG2 was conducive to the polarization of macrophages to M1 macrophages, and the polarized M1 macrophages might convert the suppressed TME to anti-tumor TME. Numerous evidences have confirmed that monocytes and the above immune cell types, as well as the presence of tertiary lymphoid structures, are associated with a good prognosis and that their infiltration into the TME is a positive prognostic indicator in many tumors.^[59–61]^ Although the ability of activated NK cells to destroy solid tumors has been questioned, their capacity to prevent metastatic dissemination by killing circulating cancer cells is well known. Some researchers showed that NK cells arrive early in the TME and cooperate with dendritic cells resulting in effective immune responses mediated by CD8T cells.^[62,63]^ In particular, PLCG2 was shown to be vital for cellular cytotoxicity in natural killer (NK) cells.^[64]^ Therefore, the higher the expression of PLCG2, the lower the resting NK cell density, which suggests that the regulation of the activity of resting NK cells might play an anti-tumor role. In recent years, clinical researchers have attempted to improve NK cell activity using various anti-tumor approaches and have decreased the number of resting NK cells, which are disadvantageous to tumor suppression.^[65,66]^ In addition, M0 macrophage infiltration in the TME has been proved to be a marker of poor prognosis in some tumors.^[14,67]^ Thus, these results indicate that the expression of PLCG2 influenced activation of the immune component within the TME in STS. PLCG2 might promote an anti-tumor TME by affecting the density, composition, and distribution of these above immune cells. But there are no corresponding drugs to modulate the activity of plcg2 clinically, which implied that there might be a unique regulatory mechanism of the role of PLCG2 in TME. While Phospholipase C (PLC), proteins encoded by PLC gene, could be inhibited by aminosteroid U73122. U73122, which could selectively inhibit a series of reactions induced by PLC via inhibiting from PLC cleaving second messengers PIP2 to IP3 and DAG, had been broadly applied to investigate PLC-dependent activation and modulation.^[68,69]^ Aki et al^[70]^ identified that increased-tyrosine phosphorylation level of PLCG2 with lipopolysaccharide (LPS) and peptidoglycan (PGN) stimulation, inducing the Ca^2+^ mobilization in macrophages and dendritic cells, enhancing cytokine production. Besides, they also found that Both PGN- and LPS-induced intracellular Ca^2+^ mobilization motioned above was not observed in PLCG2 knockout mice, implied that PLCG2 and PLCG2 signal pathway played an important role in the bacterial ligands-induced reactions in cell-mediated immunity. We believe that in-depth study of the downstream molecular mechanisms related to PLCG2 will be a direction for future research, which will provide a new perspective for further understanding of the pathogenesis of STS and the selection of diagnostic and therapeutic targets.

## 5 Conclusion

We successfully identified PLCG2 as a TME-related gene in STS via a variety of bioinformatics analysis methods. The limitations of this study include that the correlation between PLCG2 and TME status in STS was not explored completely and the sample capacity was relatively small. In conclusion, PLCG2 has potential as an important indicator of prognosis and TME remodeling in STS.

## Acknowledgments

The results of this study are based on data from TCGA (https://www.cancer.gov/tcga). We thank the authors who provided the data for this study. We thank Let Pub (www.letpub.com) for its linguistic assistance during the preparation of this manuscript.

## Author contributions

**Data curation:** Lingling Jiang, Rong Zhao, Chan Li, Wenkang Yang.

**Formal analysis:** zhengtian li, Lingling Jiang, Rong Zhao, Jun Huang, Zhenpei Wen, Gang Du.

**Funding acquisition:** Gang Du.

**Investigation:** Gang Du.

**Methodology:** Gang Du.

**Project administration:** Gang Du.

**Resources:** Gang Du.

**Software:** zhengtian li.

**Writing – original draft:** zhengtian li, Chan Li, Wenkang Yang, Jun Huang.

**Writing – review & editing:** zhengtian li, Zhenpei Wen.
